# *In vitro*study on the stability of mini-implants under simulated orthodontic loading conditions

**DOI:** 10.6026/973206300213839

**Published:** 2025-10-31

**Authors:** Kartikaya Verma, Divya Babuji Pandiyath, Ashok Panika, Shaina Goyal, Sovesh Das, Disha Pitroda

**Affiliations:** 1 Department of Orthodontics, Himachal Dental College Sundernagar, Himachal Pradesh, India; 2 Department of Orthodontics and Dentofacial Orthopedics, PSM College of dental science and Research, Thrissur, Kerala; 3 Department of Orthodontics Dentofacial Orthopedics, Government College of Dentistry, Indore, Madhya Pradesh, India; 4 Department of Orthodontics and Dentofacial Orthopedics, Bhojia Dental College and Hospital, Baddi, Himachal Pradesh, India; 5 Department of Public Health Dentistry, Kalinga Institute of Dental Scinece (KIDS), Kalinga Institute of Industrial Technology KIIT, Deemed to be University, Bhubaneswar, Odisha, India; 6 Department of Orthodontics, Karnavati University Uvarsad Gandhinagar, Gujarat, India

**Keywords:** Mini-implants, orthodontic anchorage, cyclic loading, primary stability, secondary stability, *In vitro*

## Abstract

Orthodontic mini-implants provide essential skeletal anchorage but their clinical success varies due to differences in mechanical stability under functional loading. Therefore, it is of interest to compare the stability of three commercially available designs-dual- thread, single-thread and tapered-using synthetic bone blocks. Primary stability was assessed by insertion torque and resonance frequency analysis, followed by cyclic loading to evaluate secondary stability, displacement and failure rates. Dual-thread implants demonstrated superior performance with higher stability, lower displacement and fewer failures compared to the other designs. Thus, we show that implant design plays a critical role, with dual-thread configurations offering clear clinical advantages for anchorage-dependent orthodontic treatments.

## Background:

Orthodontic mini-implants have revolutionized skeletal anchorage by enabling non-compliant tooth movement and complex biomechanics [1]. Despite their widespread adoption, clinical failure rates ranging from 10% to 30% remain a significant concern, often attributed to inadequate stability under functional loads [2]. Stability is governed by primary (mechanical interlock at insertion) and secondary (biological integration) phases, both influenced by implant design, bone quality and loading protocols [3]. Recent clinical studies highlight the correlation between primary stability metrics-insertion torque (IT) and resonance frequency analysis (RFA)-and long-term success [4]. However, most investigations focus on static or short-term loading, neglecting the dynamic cyclic forces inherent in orthodontics [5]. Cyclic loading induces microdamage at the bone-implant interface, potentially leading to secondary instability and failure [6]. While finite element analyses suggest design-specific stress distributions, empirical *in vitro* data under simulated orthodontic conditions are scarce [7]. A critical research gap exists in quantifying how thread geometry and taper affect stability during prolonged cyclic loading. Prior studies primarily compare single versus dual-thread designs in static models, with limited emphasis on displacement and failure under fatigue conditions [8]. Therefore, it is of interest to address this gap by evaluating three distinct mini-implant designs under standardized cyclic loading, providing clinically relevant insights into mechanical performance. Hence the aim was to investigate the primary and secondary stability of dual-thread, single-thread and tapered mini-implants under simulated orthodontic cyclic loading and to correlate design features with displacement and failure rates.

## Materials and Methods:

## Study design:

*In vitro* experimental study comparing three mini-implant groups.

## Sample size:

Sixty mini-implants (n = 20 per group) were selected based on power analysis (α = 0.05, β = 0.2, effect size = 1.2).

## Inclusion criteria:

[1] Unused titanium alloy mini-implants (grade 5).

[2] Diameter: 1.6 mm; length: 8 mm.

[3] Three designs:

1) Group A: Dual-thread (Jeil Medical, Seoul, Korea).

2) Group B: Single-thread (Forestadent, Pforzheim, Germany).

3) Group C: Tapered (HDC, Maccagno, Italy).

## Exclusion criteria:

[1] Implants with manufacturing defects.

[2] Non-standard dimensions.

## Materials:

[1] Synthetic bone blocks (Sawbones, Pacific Research Labs, USA): Cortical layer (2 mm, density 40 PCF) over cancellous bone (20 PCF).

[2] Torque wrench (Tohnichi, Tokyo, Japan) for IT measurement.

[3] Osstell ISQ device (Integration Diagnostics, Sweden) for RFA.

[4] Cyclic loading machine (Instron 5566, USA) with 100-N load cell.

[5] Digital micrometer (Mitutoyo, Japan; accuracy ±0.001 mm).

## Procedures:

[1] Insertion: Mini-implants inserted perpendicular to the bone surface at 30 rpm. IT recorded during placement.

[2] Primary stability: RFA measured immediately post-insertion (two perpendicular readings; average used).

[3] Cyclic loading: Samples subjected to 50,000 cycles of 2-N force at 2 Hz (simulating 6 months of orthodontic force).

[4] Secondary stability: RFA repeated post-loading.

[5] Displacement: Linear displacement measured at the implant head using a micrometer.

[6] Failure criteria: Implant fracture, displacement >0.5 mm, or visible loosening.

## Statistical analysis:

[1] Data expressed as mean ± standard deviation (SD).

[2] One-way ANOVA with Tukey post-hoc test for intergroup comparisons.

[3] Chi-square test for failure rates.

[4] Significance: p < 0.05 (SPSS v26.0, IBM).

## Results:

Group A exhibited significantly higher IT (15.2 ± 1.8 Ncm) and RFA (68.5 ± 3.2 ISQ) than Group B (12.8 ± 1.5 Ncm; 62.3 ± 2.8 ISQ) and Group C (10.5 ± 1.2 Ncm; 58.7 ± 3.1 ISQ; p < 0.001). Post-loading, Group A maintained superior RFA (65.2 ± 3.5 ISQ) and minimal displacement (0.12 ± 0.03 mm). Group B and C showed progressive stability loss and increased displacement (p < 0.001; Table 1 - see PDF). Group A had the lowest failure rate (5%), followed by Group B (10%) and Group C (20%; p = 0.008; Table 2 - see PDF). IT strongly correlated with RFA pre-loading (r = 0.89, p < 0.001) and post-loading displacement (r = -0.82, p < 0.001) (Table 3 - see PDF).

## Discussion:

Mini-implants have gained wide acceptance as reliable temporary anchorage devices in orthodontics, yet their reported success rates vary between 70-95% depending on patient- and device-related factors [1]. Primary stability, achieved at insertion, is largely dependent on mechanical interlocking with cortical bone, while secondary stability develops through bone remodeling and adaptation [2]. *In vitro* models provide a controlled environment to assess the mechanical contributions of implant geometry, thread design and cortical engagement without the confounding effects of biological healing [3]. Thread morphology has been recognized as a critical determinant of stability, with deeper and dual-thread configurations demonstrating superior bone contact ratios compared to single-thread or tapered forms [4]. Studies using pull-out and torque resistance tests confirm that dual-thread designs enhance mechanical anchorage by increasing surface area and reducing microstrain concentrations [5]. This aligns with clinical findings where thread pitch and depth significantly influence survival rates under orthodontic forces [6]. Cyclic loading simulations, as applied in the present study, replicate the repetitive nature of orthodontic forces and allow evaluation of fatigue resistance over time [7]. Such dynamic models are essential, as static insertion torque values alone may overestimate long-term stability. By subjecting implants to 50,000 loading cycles, we approximate masticatory and orthodontic stresses that implants endure over several months, thereby providing insight into early mechanical failure mechanisms before biological adaptation occurs. This study demonstrates that mini-implant design critically influences stability under cyclic orthodontic loading. Group A (dual-thread) exhibited superior primary and secondary stability, minimal displacement and the lowest failure rates, aligning with finite element analyses showing optimized stress distribution in dual-thread designs [9]. The higher IT and RFA in Group A likely reflect enhanced mechanical interlock due to increased thread surface area and cortical bone engagement [10]. Post-loading stability loss was most pronounced in Group C (tapered), corroborating clinical reports of higher failure rates in tapered implants under dynamic loads [11]. Tapered designs concentrate stress at the implant neck, accelerating microdamage and displacement [12]. In contrast, dual-thread implants distribute forces more evenly, mitigating interface degradation [13]. The strong correlation between IT and post-loading displacement (r = -0.82) underscores primary stability as a predictor of long-term performance, consistent with clinical studies [14]. The 20% failure rate in Group C exceeds typical clinical ranges (10-30%), potentially due to the absence of biological healing in our model [15]. However, this highlights the vulnerability of tapered designs to mechanical fatigue. Our cyclic loading protocol (2 N, 50,000 cycles) approximates 6 months of orthodontic force, providing clinically relevant insights into early failure mechanisms [16]. Limitations include the use of synthetic bone, which cannot replicate biological remodeling or inflammatory responses. Future studies should incorporate dynamic fluid environments and osteoblast-seeded scaffolds to simulate biological integration [17].

## Conclusion:

Mini-implant design significantly impacts stability under orthodontic loading. Dual-thread implants demonstrate superior primary and secondary stability, reduced displacement and lower failure rates compared to single-thread and tapered designs. These findings advocate for design selection based on mechanical performance to enhance clinical success in anchorage-dependent orthodontic treatments.
